# No effect of intradialytic neuromuscular electrical stimulation on inflammation and quality of life: a randomized and parallel design clinical trial

**DOI:** 10.1038/s41598-021-01498-7

**Published:** 2021-11-12

**Authors:** Ana C. B. Marini, Reika D. Motobu, Patrícia C. B. Lobo, Paula A. Monteiro, Gustavo D. Pimentel

**Affiliations:** 1 Faculty of Nutrition, Federal University of Goias, Rua 227, Quadra 68 s/n°, Setor Leste Universitário, Goiânia, GO CEP: 74605080 Brazil; 2 Immunometabolism Research Group, Department of Physical Education, São Paulo State University, Presidente Prudente, SP Brazil

**Keywords:** Nephrology, Kidney, Kidney diseases, Renal replacement therapy

## Abstract

Neuromuscular electrical stimulation (NMES) elicits muscle contraction and has been shown to improvement of quality of life. However, if NMES improvement the quality of life and attenuate the inflammation is not fully understood. Therefore, our aim sought to assess the effects of short-term of intradialytic NMES on inflammation and quality of life in patients with chronic kidney disease patients undergoing hemodialysis. A randomized clinical trial conducted with parallel design enrolled adult hemodialysis patients three times a week during 1 month. Patients were randomly assigned to two groups (control group, n = 11; 4F/7 M) or (NMES group, n = 10; 4F/6 M). Pre-and post-intervention, was measured the high-sensitivity C reactive protein, interleukin-6, interleukin-10, and TNFα by the ELISA, and quality of life was applied using the SF-36. During each hemodialysis session, NMES was applied bilaterally at thigh and calves for 40 min. There was not change in cytokines (hs-CRP, IL-6, IL-10, and TNFα) concentrations time × group interaction. In addition, no difference was found in eight domains of quality of life. In addition, the groups did not differ for muscle strength and muscle mass. In conclusion, we found that intradialytic NMES did not change inflammation neither quality of life.

## Introduction

Patients with chronic kidney disease (CKD) undergoing hemodialysis (HD) experiences increase of inflammatory cytokines that are associated with the diminished appetite^1^ and reduced physical mobility which leads to significant loss of quality of life (QoL)^2–5^. The persistent inflammation in HD is multifactorial a clinic complication and its mechanisms are still unclear, believes that it has relation with some exogen factors like (dialysis membranes and central venous catheters), cellular (oxidative stress and cellular aging), tissue factors (hypoxia, fluid and sodium overload), microbiological (immune dysfunction and intestinal dysbiosis) and uremic toxin retention such as indoxyl sulphate, advanced glycation end products and calcioprotein particles^6^.

Indeed, physical activity is crucial to attenuate the inflammatory response and QoL loss. However, for many of HD patients, the physical activity is not feasible and tolerated. Thus, neuromuscular electrical stimulation (NMES) has been used during the disuse to maintenance of anabolism^7^ and improvement of QoL.

In previous studies, our group shown that after one month of supplementation with creatine associated with NMES in HD patients, there was improvement in the fields of vitality, physical function and emotional aspects of SF-36 questionnaire^8^. This study showed correlation between physical role functioning and leg extension one-repetition maximum (1RM) at post-intervention when compared to pre-intervention, but there was no correlation between the physical role functioning and handgrip strength^8^. We also demonstrated in a pilot study with 21 patients that isolated intradialitic electrical stimulation for one month promoted an improvement in the phase angle, however there was no change in lean body mass^9^.

Although, the intradialytic NMES had improved the QoL^10,11^, no studies assessed its effects on inflammatory profile. Therefore, we hypothesized that intradialytic NMES would attenuate the inflammation and improvement the QoL in patients undergoing HD. Therefore, our aim sought to assess the effects of short-term of intradialytic NMES on inflammation and QoL in CKD patients undergoing HD.

## Methods

### Study design and patients

A randomized design parallel groups study was performed in CKD patients undergoing HD. This trial was approved by the local ethical committee (Federal University of Goias under the number 1.470.351), informed consent was obtained from all patients and registered in the Brazilian Registry of Clinical Trials (REBEC) under the code RBR-98wzgn. All methods were performed in accordance with the relevant guidelines and regulations.

**Figure 1:**
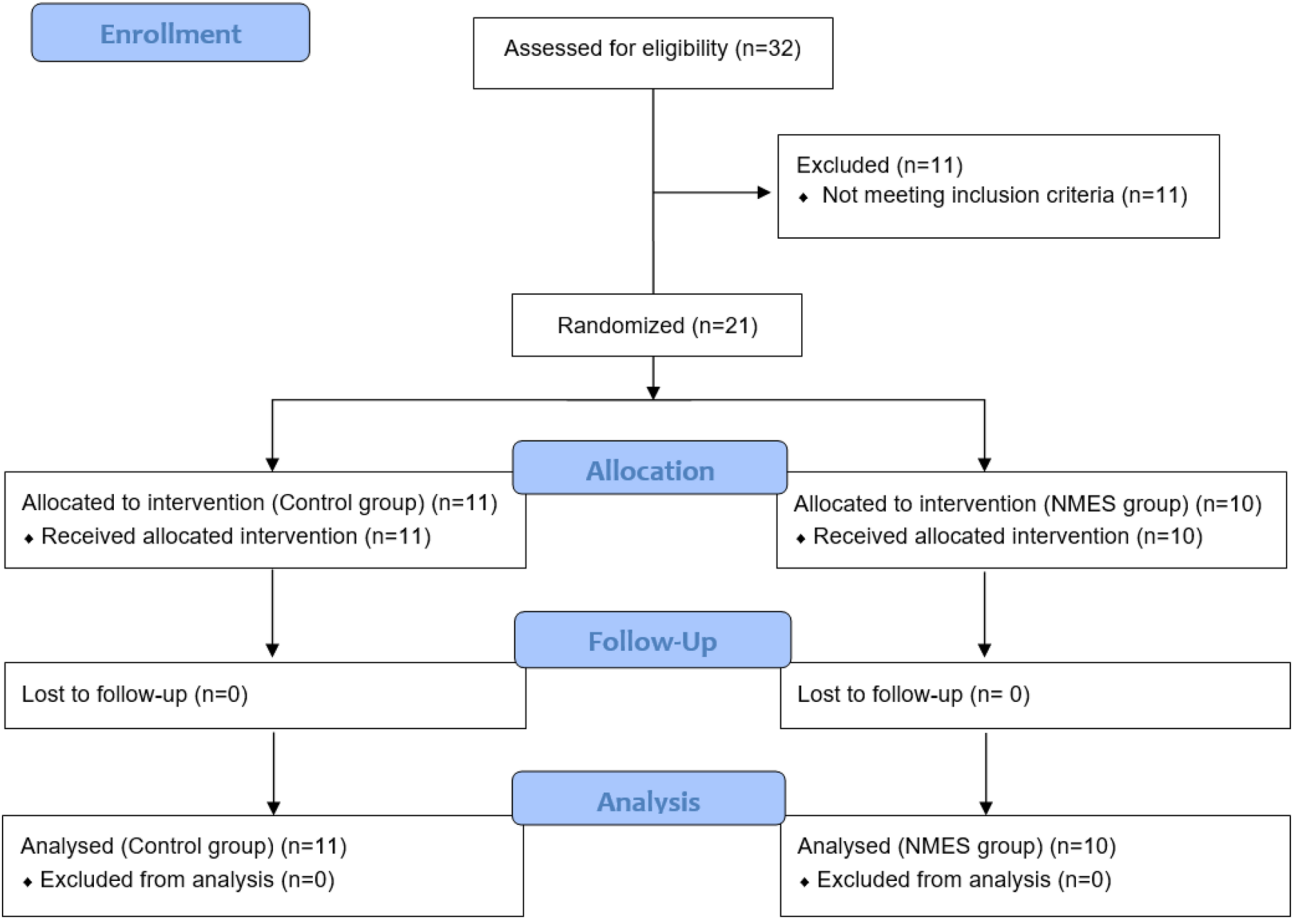
Consort flowchart. *NMES* Neuromuscular electrical stimulation.

The sample was composed of outpatients with CKD undergoing HD for more than 3 months and aged 18 years recruited from two HD clinics. The exclusion criterion were overweight patients, neurological disease, severe cardiovascular diseases, physical impairments (amputations, deep vein thrombosis), in use of oral nutritional supplements or recent change in food intake and patients who underwent structured physical training 3 months prior to the date of inclusion in the study. The inclusion criteria were patients undergoing HD for a minimum of 3 months, as previously demonstrated^9^. Of the 32 eligible patients, 11 were excluded because they were overweight and obese and a total of 21 patients were randomized between groups control (Control group, n = 11) or intervention (NMES group, n = 10) (Fig. 1).

The study consisted of 6 weeks of duration and in the 1st week, the initial assessments were carried out with the application of socioeconomic questionnaires, SF36, physical evaluation (bioimpedance analysis and handgrip strength) and blood collection. In the 2nd to the 5th week, interventions were performed with the NMES and in the 6th week, the intervention was completed within a maximum period of 48 h after the last NMES session and during the second session (intermediate session) of HD, these patients were again assessed with application of the SF36 questionnaire, physical evaluation and biochemical exams.

### Intervention with neuromuscular electrical stimulation

The intervention was based on our previous study in adult^9^. Both groups received usual care, being that the control group did not receive training and the NMES received the intradialytic electrical stimulation for 40 min three times per week during one month. The protocol consisted of three phases: 1st) ‘warming up’ for five minutes at a frequency of 5 Hz, 250 μs (continuous mode); 2nd) muscle stimulation for 30 min, with frequency of 100 Hz, 400 μs (burst/contraction mode) and 3rd) relaxation for five minutes at a frequency of 5 Hz, 250 μs (continuous mode). The electrodes were applied on the right and left regions on the vastus lateralis quadriceps muscle of thighs and on the gastrocnemius muscle of calves using the TENS (CARCI, São Paulo, Brazil, Model TENSMED IV-4034) and following the protocol of previous study^9,12^. Therefore, eight self-adhesive electrodes 5 × 5 cm were attached which provided the distribution of the electrical current and so that both legs receive the stimulus simultaneously. The stimulus amplitude was increased until the visible contraction. During the NMES sessions, patients were encouraged to increase the potency of the stimulus relative to the previous session according to their tolerance. The control of intensity/frequency was done by trained researchers. At the end of each session, the points of electrical stimulus in thighs and calves were remarked with permanent marker so that in all sessions were done in the same place. Previous studies have considered NMES safe and without side effects^9,13^.

### Quality of life assessment

The QoL was assessed by SF-36 questionnaire applied before and after of the intervention. It contains 36 items and eight domains summarized in two dimensions, physical and mental health. It is a well-documented questionnaire that has been widely used. The eight domains of SF-36 are summarized in two dimensions: physical health and mental health^14^.

### Bioimpedance analysis

The bioimpedance analysis (BIA) was performed with the Biodynamics device (Model—450A, Bioimpedance Analyzer, Seattle, WA, USA), half an hour after the intermediate HD session of the week, with the patients lying horizontally. Muscle mass was quantified by the equation developed by Janssen et al. and recommended by the “European Working Group on Sarcopenia in Older People (EWGSOP)”^15,16^.

### Handgrip strength (HGS) assessment

HGS was performed on the dominant limb that did not have an arteriovenous fistula in use, using a portable hydraulic dynamometer (CAMRY, China). The patient should be in a standing position with the arm at a 90° angle to the trunk. The measurement was performed in duplicate with a difference of at least five seconds between repetitions, so the highest value of the results was adopted^17^.

### Biochemical analyses

Blood collection was performed by trained professional, after the second session of HD (intermediary session) and 48 h after the last intervention with NMES. Immediately after punction blood, it was centrifuged at 4000 rpm for 10 min at 4 °C (Hitachi CF16RN, Ibaraki, Japan) and stored at − 80 °C for later quantification of cytokine concentrations. High-sensitive-CRP was quantified using the chemiluminescent immunoassay method (ARCHITECT c8000, Abbot Park, Illinois, EUA) and the interleukins and TNFα were analyzed using the human ELISA Ready-Set-Go kits (eBioscience® Vienna, Austria).

### Statistical analyses

For the sample size calculation, was calculated as described previously^9^. The analysis data were performed using the Statistical Package of Social Sciences (SPSS) version 18.0 and R Studio version 3.4.3 programs. Descriptive statistics (frequencies, means and standard error of the mean) were obtained. Numerical variables were tested for normality by the Kolmogorov–Smirnov test. Two-way ANOVA test was done to evaluate the interaction between time (pre and post) and treatment (NMES or control). For those significant statistically variables, the effect size (d, Cohen) were performed. Difference statistical was set at 5%.

## Results

The study consisted of twenty-one adult (41.7 ± 10.8 years) patients (men = 13, 61.9% and female = 8, 38.1%) divided into two groups: control (n = 11, 7 M and 4F) and NMES (n = 10, 6 M and 4F). The groups did not differ for age (Control group 45.8 ± 10.8 years, NMES 37.3 ± 9.2 years, *p* = 0.07), sex (*p* = 0.86) and BMI (*p* = 0.15). In addition, the groups did not differ for muscle strength (Control group: pre 28.6 ± 7.6 vs. post 30.5 ± 8.8 and NMES 32.5 ± 12.8 vs. post 33.7 ± 12.9, interaction time × group *p* = 0.57) and for muscle mass (Control group: pre 25.2 ± 4.7 vs. post 24.1 ± 3.1 and NMES 25.2 ± 2.7 vs. post 26.2 ± 3.0, interaction time × group *p* = 0.06). The weekly percentual incremental of the NMES in each phase was showed in Supplementary Fig. 1. We did not find change in cytokines profile (hs-CRP, IL-6, IL-10 and TNFα) concentrations in interaction time × treatment. Despite the difference in group for the TNFα levels and pain domain, the post-hoc and sample pairs analysis did not detect difference (Tables 1 and 2).

**Table 1:** Biochemical analyses.

Parameters	Control		NMES		Effect size	*p* group	*p* time	*p* interaction
	Pre	Post	Pre	Post				
	ME ± SD (95%CI)	ME ± SD (95%CI)	ME ± SD (95%CI)	ME ± SD (95%CI)				
Hs-CRP (mg/dL)	13.9 ± 36.5 (10.6;38.5)	2.3 ± 1.9 (1.0;3.6)	20.3 ± 25.7 (1.0;3.6)	10.5 ± 14.0 (0.4;20.5)	0.006	.257	.100	.888
Interleukin 6 (pg/mL)	5.0 ± 5.0 (1.4;8.6)	3.4 ± 1.4.1 (0.2;6.2)	11.0 ± 27.8 (0.2;6.2)	6.1 ± 14.7 (− 4.3;16.6)	18.179	.424	.544	.810
Interleukin 10 (pg/mL)	3.5 ± 5.6 (− 0.6;7.4)	2.0 ± 2.4 (0.1;3.6)	2.3 ± 2.9 (0.1;3.6)	0.9 ± 0.5 (0.5;1.3)	32.301	.154	.359	.587
TNFα (pg/mL)	14.7 ± 13.7 (3.8;24.9)	18.0 ± 22.6 (− 0.8;36.9)	6.8 ± 10.1 (− 0.8;36.9)	4.5 ± 2.9 (2.3;6.6)	32.475	.015	.918	.481

ANOVA two-way (group × time interaction).

*ME* Mean, *SD* Standard deviation, *Hs-CRP* High sensitive C reactive protein, *TNFα* Tumor necrosis factor alpha, *NMES* Neuromuscular electrical stimulation.

Regarding to QoL, we also did not observe difference in any of eight domains evaluated (Table 2).

**Table 2:** Quality of life SF-36.

Parameters	Control		NMES		Effect size	*p* group	*p* time	*p* interaction
	Pre	Post	Pre	Post				
	ME ± SD (95%CI)	ME ± SD (95%CI)	ME ± SD (95%CI)	ME ± SD (95%CI)				
Physical function	76.3 ± 22.2 (61.4;91.3)	73.1 ± 44.7 (43.0;103.2)	80.5 ± 24.7 (62.7;98.2)	77.0 ± 24.4 (59.5;94.4)	0.029	.627	.683	.984
Physical role functioning	40.9 ± 37.5 (15.6;66.1)	68.1 ± 65.2 (24.3;112.0)	57.5 ± 40.9 (28.2;86.7)	82.5 ± 47.2 (48.7;116.2)	0.114	.294	.083	.937
Pain	55.5 ± 28.0 (36.7;74.3)	59.4 ± 33.6 (36.8;82.0)	67.0 ± 31.2 (44.6;89.7)	79.3 ± 28.5 (58.8;99.7)	0.834	.041	.283	.564
General health perceptions	50.6 ± 20.9 (36.5;64.7)	43.0 ± 29.2 (23.3;62.6)	52.5 ± 18.7 (39.0;65.9)	43.9 ± 18.4 (30.6;57.1)	0.097	.849	.274	.947
Vitality	56.3 ± 20.3 (42.6;70.0)	53.6 ± 28.1 (34.7;72.5)	56.0 ± 22.0 (40.2;71.7)	61.5 ± 14.5 (51.0;71.9)	0.906	.576	.858	.539
Social role functioning	69.3 ± 24.5 (52.7;85.8)	78.4 ± 36.2 (54.0;102.4)	83.7 ± 25.0 (65.8;101.6)	86.2 ± 23.1 (69.6;102.8)	0.883	.140	.421	.654
Emotional role functioning	51.5 ± 47.9 (19.2;83.7)	57.5 ± 66.8 (12.6;102.4)	53.3 ± 50.1 (17.4;89.2)	70.0 ± 42.8 (39.3;100.6)	0.583	.588	.401	.686
Mental health	72.0 ± 24.1 (55.7;88.2)	63.6 ± 34.0 (40.7;86.5)	61.2 ± 25.9 (42.6;79.7)	69.6 ± 25.8 (51.0;88.1)	2.570	.614	.936	.092

ANOVA two-way (group × time interaction).

*ME* Mean, *SD* Standard deviation, *NMES* Neuromuscular electrical stimulation.

## Discussion

To the best of the author’s knowledge, this study to assess the effects of intradialytic NMES on inflammatory cytokines and QoL. Additionally, intradialytic NMES targeting the leg muscles did not change the inflammation and QoL in patients undergoing HD. Therefore, different from our hypothesis, the inflammation is not impacted by NMES.

Regarding inflammation, the literature points to the need to practice intense exercises such as high intensity resistance exercise > 60–70% of one repetition maximum (RM)^18^ or physical activities that reach more than 70% of the pulmonary O2 uptake peak (Vo2.peak)^19^ to reduce oxidative stress by increasing concentrations of anti-inflammatory cytokines^20^.

Muscle contractions promote the production of IL-6 which may be involved in muscle repair and may have anti-inflammatory effects through the induction of the expression of the IL-10 receptor^20,21^. The NMES has been compared to physical activity and evidence shows that when performed throughout the body (at frequencies between 50 and 90 Hz) it has no difference for the increase in muscle strength^22^ and the gain in lean mass^23^ when compared to activities of medium to high intensity as in resistance training and high intensity interval training. Thus, we believed that NMES, as well as resistance training, are able to stimulate anti-inflammatory cytokines. However, in the present study, even that NMES was applied both legs of the vastus lateralis muscle of the quadriceps with frequency of 100 Hz we did not confirm this hypothesis. In this sense, the effectiveness of NMES in controlling the inflammatory response is questioned, as this is a modality comparable to the practice of light exercises^24^. In ten healthy adults of both sexes, after a single session of 50 Hz NMES for 30 min, an increase in peak IL-6 concentration was noted between 30 and 120 min after stimulation and a reduction in minimum IL-1 values after 30 min of stimulation^24^. Although, Carrero et al. observed in HD patients that IL6 levels were not different between sex, suggesting that there is no influence of this factor for changes in inflammatory cytokines^25^, further studies are warranted to evaluate the effects of NMES on inflammatory profile.

In this sense, we found a single study carried out by Brüggemann et al., which stimulated HD patients with NMES and evaluated inflammatory markers^26^. The frequency of 50 Hz was applied for 60 min, three times a week for 1 month. In this work, unlike our study in which there were no changes, they observed a reduction in the isolated concentration of IL-10. However, since this is an isolated alteration, this result must be evaluated with caution, since the modification of a single cytokine, in addition to not having a significant impact on inflammation, does not allow conclusions to be reached regarding the effect of NMES on the inflammatory process, which is controlled by a wide and complex network of cytokines that act locally or systemically. Thus, it is not yet clear whether the NMES performed during the HD process can reduce the inflammatory process.

It has been reported that 12–20 weeks of intradialytic NMES improves components of the QoL as physical or mental^10,11^. So, as our intervention lasted only 4 weeks, we believe that the time was not enough to promote significant changes in the perception of patients, as observed in the study by Suzuki et al. (2018)^27^, which lasted 8 weeks and although they observed improvement in the components of the SF-8-china QoL questionnaire, there was no statistical difference. In addition, the lack of standardization in the QoL questionnaire used, EuroQol-5D^11^ and SF8-china^27^ makes it difficult to compare the findings.

Another important aspect is the non-standardization of the NMES protocol used, which varied the intensity applied from 10 Hz for 60 min^10^ to 90 Hz for 38 min^11^. Our study also did not carry out previous sessions of muscle adaptation and toning as performed by Simó et al. that improve functional capacity and QoL of their patients on HD^11^.

Additionally, studies with longer duration with the exercise may help in clinical practice, favoring health professionals to promote greater independence and agility of patients, reducing the fragility and sarcopenia-related QoL. Despite the lack of results in our study, because it is a practical, fast and low-cost methodology, the use of NMES could be included in the HD routine of patients as a muscle movement strategy^10^.

As strengths of our study, we highlighted: (i) We evaluate cytokines as markers of inflammation. However, some limitations are quite important and further studies are warranted, such as (i) the duration of the intervention with NMES and (ii) standardization of the NMES protocol.

In conclusion, we found that intradialytic NMES did not change inflammatory profile neither QoL.

## Supplementary Information

Supplementary Information.
