# Supplementary material for: No effect of intradialytic neuromuscular electrical stimulation on inflammation and quality of life: a randomized and parallel design clinical trial
Source: Sci Rep. 2021 Nov 12;11:22176. doi: 10.1038/s41598-021-01498-7 (PMC8590010; doi:10.1038/s41598-021-01498-7)
Supplement: Supplementary file 1 — Supplementary Information. [file 41598_2021_1498_MOESM1_ESM.docx]

**
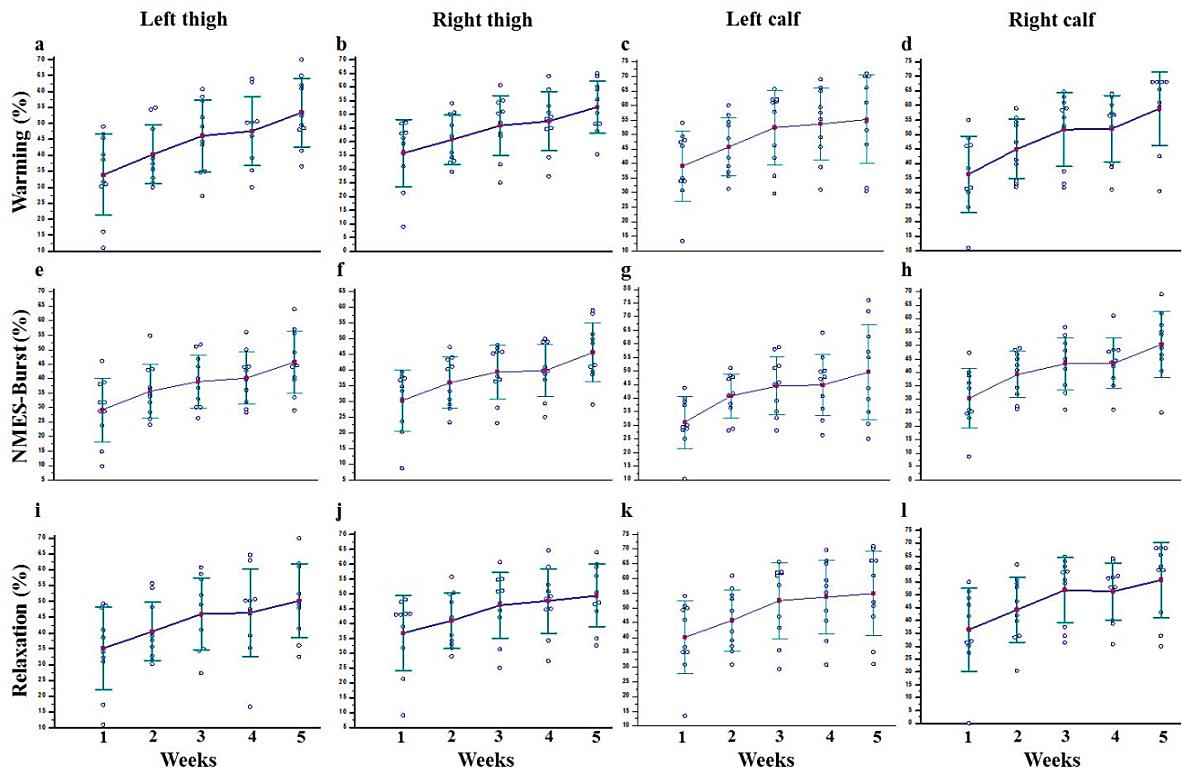
**

**Supplementary Figure 1.** Evolution of NMES during the intervention. Data are shown in ME±SEM. NMES: Neuromuscular electrical stimulation; ME: average; SEM: mean standard error.
